# Phytochemical analysis and nephroprotective potential of Ajwa date in doxorubicin‐induced nephrotoxicity rats: Biochemical and molecular docking approaches

**DOI:** 10.1002/fsn3.3199

**Published:** 2023-01-27

**Authors:** Othman A. S. Baothman, Hisham N. Altayb, Mustafa A. Zeyadi, Salman B. Hosawi, Mohamed Kamel Abo‐Golayel

**Affiliations:** ^1^ Biochemistry Department, Faculty of Science King Abdulaziz University Jeddah Saudi Arabia; ^2^ Microbial Toxicology & Natural Products Center, Faculty of Science King Abdulaziz University Jeddah Saudi Arabia; ^3^ Ain Shams Medical Research Center, Faculty of Medicine Ain Shams University Hospitals, Ain Shams University Cairo Egypt

**Keywords:** Ajwa date, doxorubicin, molecular docking, nephroprotective, nephrotoxicity, phytochemical

## Abstract

The purpose of this study is to evaluate the likely defensive impact of Ajwa date aqueous extract (AJDAE) in alleviating the nephrotoxicity generated by doxorubicin (DOX) injection in rats. Sixty male Wister albino rats were randomly and equally separated into six groups (*n* = 10), and they were treated as follows: untreated control group, extract groups administered with 0.75 and 1.5 mg kg bw of AJDAE, toxicant control group administered with DOX, and prophylactic groups were treated with 0.75 and 1.5 mg/kg of AJDAE and 15 mg/kg DOX. Biochemical parameters, antioxidant enzymes, renal functions, DNA integrity, and histopathology were studied to evaluate the nephroprotective activity of AJDAE. Furthermore, bioactive compounds were utilized for in silico molecular docking. AJDAE treatment resulted in significant improvements in the amended renal biomarkers (urea, creatinine, calcium, phosphorous, and uric acid), antioxidative markers, and MDA. Noticeable histopathological improvements supported this result. Results of in silico studies revealed that d‐Mannitol, 6TMS derivative, palmitic acid, and TMS derivative had a higher docking score with human soluble epoxide hydrolase (−10.9 kcal/mol) and NF‐κB‐DNA (−7 kcal/mol). The present findings indicated that AJDAE could decrease ROS generation and lipid peroxidation (LPO) and repair the DOX injection‐related DNA damage.

## Graphical abstract

The nephroprotective effect of the Ajwa date extract could be attributed to its potential antioxidant effect that contains phytochemicals with potential therapeutic value for the nephrotoxicity.

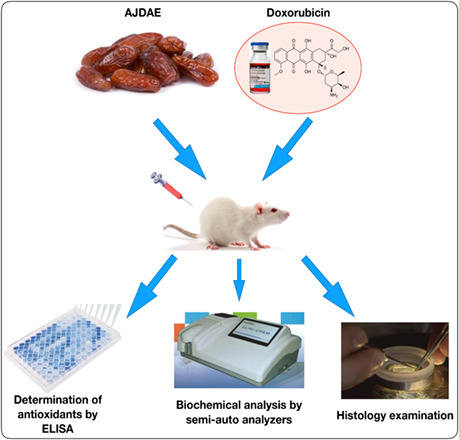

## 1 INTRODUCTION

Increasing in prevalence globally, chronic kidney disease is a significant public health concern that can potentially result in kidney failure. Kidney disease entails a progressive reduction in kidney function attributable to nephron decreases (Hrenák et al., 2013). Although it has strong antitumor effectiveness, doxorubicin (DOX) is limited as a chemotherapeutic drug due to its cardiac, pulmonary, testicular, and renal toxicity (Fadillioǧlu et al., 2003; Sabbah et al., 2018). In chronic kidney disease animal models, DOX‐induced nephrotoxicity is renowned and widespread; its detrimental impact is managed through proximal tubules' discriminatory cellular injury via mechanisms that stay as the core (Grant et al., 2019). Despite doxorubicin's acute cellular toxicity mechanism ambiguity, ROS is a primary element of DOX toxicity; thus, the factors that manage this oxidative harm are comprehensively researched (Reddy et al., 2007). DOX injection produces hydrogen peroxide, superoxide anions, and hydroxyl radicals. DOX is transformed into a semiquinone free radical by NADPH‐cytochrome P‐450, which then initiates superoxide anion and hydroxyl radicals' generation, producing LPO (Oz & Ilhan, 2006). Although many studies have examined the DOX‐initiating molecular mechanism, there are gaps surrounding its causal factors. Possibly, the basis of this is DOX producing DOX semiquinone. While the resultant semiquinone radical does not have a long life span, it creates a torrent of reactions producing ROS upon reacting with O_2_ ROS (El‐Shitany et al., 2008; Liu et al., 2007; Mohan et al., 2010; Rašković et al., 2011). Evidently, using doxorubicin results in improved production of superoxide molecules, hydrogen peroxide, and hydroxyl radicals, which can rapidly interact with membrane lipids, resulting in LPO (Oz & Ilhan, 2006). LPO is a core component of the toxic indicators of DOX administration and is assessed in line with MDA intensities. In rats, DOX‐induced nephrotoxicity exhibits severe renal LPO (Akyol et al., 2014; Yagmurca et al., 2004).

Date palm fruits (DPFs) are eaten in numerous countries and are particularly important in Middle Eastern and North African nations. DPF extracts contain many pharmacological properties, including antiallergic (Karasawa & Otani, 2012), antibacterial (Zhang et al., 2013), antifungal (Boulenouar et al., 2011), antioxidants (El Arem et al., 2014), anticancer (Karasawa & Otani, 2012), anti‐inflammatory (Borochov‐Neori et al., 2015), antimicrobial (Mahdhi et al., 2013), cardioprotective (Mubarak et al., 2018a; Sabbah et al., 2018), immune‐boosting (Karasawa et al., 2011), nephroprotective (Wang et al., 2019), and neurologically protective (Pujari et al., 2011). Additionally, research has shown that DPF extract can essentially hunt for oxidants due to its antioxidants and antimutagenic compounds (Allaith, 2008; Shireen et al., 2008).

Recently, natural antioxidant consumption has generated vast interest as a means of averting oxidative harm in numerous oxidative stress‐related diseases. In nephrotic disease, consumption of medicinal plant nutrients in conventional herbal medicine provides therapeutic benefits. This study was conducted to explore the possibility of prophylactic effect of AJDAE in improving doxorubicin‐induced nephrotoxicity in Wister albino rats. Furthermore, molecular docking was used to identify and compare the interaction between the active compounds and the observed biological activity through molecular docking.

## 2 MATERIALS AND METHODS

### 2.1 Ajwa date (*Phoenix dactylifera* L.) aqueous extract preparation

Ajwa date was bought from the date market in Jeddah, KSA; established and recognized by a professor of plant taxonomy; and banked in the Herbarium of Biological Sciences Department, KAU (specimen voucher number: *P. dactylifera* L. #PD17569). The flesh of Ajwa date (10 g) was drenched in 100 ml of double distilled water for 18 h and mixed up in a blender at a room temperature (22–25°C) (Abdelaziz & Ali, 2014). Ajwa date aqueous extracts were sieved and centrifuged (Hettich ZENTRIFUGEN D‐7200 Tuttlingen Type 1200 220V Benchtop Centrifuge) at 4°C and 5000 *g* for 15 min. The supernatants were withdrawn and stored at 4°C till usage, and the precipitant was removed. Fresh AJDAE was prepared daily throughout the experiment days (Vayalil, 2002) just prior oral administration by using intragastric gavage throughout the progress of the experimentation.

### 2.2 Chemicals

Doxorubicin vial was obtained from EBEWE Pharma Ges.m.b.H “Ebewe” (50 mg/25 ml Nfg. KG, A‐4866Unterach). Urea (URE120240), creatinine (CRE106240), calcium (CAL103120), phosphorous (PH123100), and uric acid (UA121240) were obtained from EGY CHEM LAB Technology. Superoxide dismutase, glutathione peroxidase, glutathione‐S‐transferase, glutathione reductase, catalase, and malonaldehyde competitive enzyme immunoassay technique were purchased from Bioassay Technology Laboratory, Shanghai Korain Biotech Co. DNA tissue extraction kit was provided by Qiagen, DNeasy, RNeasy, QIAGEN Group. Top Vision Agarose‐R0491, PBS‐1314‐87‐0, EDTA‐E1161, Tris Acetate‐EDTA buffer (TEA‐T8280), hematoxylin, and eosin were supplied from Merck, Sigma‐Aldrich.

### 2.3 Chromatographic analysis of Ajwa date using GC‐MS

Gas chromatography–mass spectrometry (GC‐MS) chromatographic analysis of Ajwa date was performed using Agilent Technologies 7890B GC Systems combined with 5977A Mass Selective Detector. Capillary column (HP‐5MS Capillary; 30.0 m × 0.25 mm ID × 0.25 μm film) and helium as a carrier gas with a rate of flow of 1.7 ml/min with 1 μl injection were also used. Analysis of the sample was carried out withholding the column initially for 4 min at 40°C postinjection, and then the temperature was elevated to 300°C (20°C/min heating ramp) along with a 3.0‐min hold. The injection was done in split‐less mode at 300°C. MS scan range was (m/z) 50–550 atomic mass units under electron impact ionization (70 eV).

### 2.4 Silylation agent: BSA N, O‐Bis (trimethylsilyl) acetamide

The reaction was carried out by adding 100 μl of BSA plus amount of the sample after extraction and heating in water bath at 70°C for 2 h and then injected into GC‐MS under the above conditions. The constituents were determined by mass fragmentations with the NIST mass spectral search program for the NIST/EPA/NIH mass spectral library (June 2014).

### 2.5 Experimental design

This study was approved by the Research Ethics Committee (REC), Faculty of Medicine, King Abdul Aziz University (KAU). Two‐month‐old male Wistar rats (150–200 g), which were bred in the animal house of King Fahd Medical Research Center (KFMR), King Abdulaziz University, KSA, were accommodated in an experimental animals care facility, including room temperature (25 ± 1°C), 12‐h light/dark cycles, and suitable humidity, and they were allowed free path to a standard pellet diet and tap water. Prior starting the experiment, rats were kept for 7 days to familiarize the surrounding environment in stainless‐steel mesh‐covered plain polypropylene cages. The experiment was approved, and the rats had animal carefulness according to the guidelines of the Committee for the Purpose of Control and Supervision of Experiments on Animals (CPCSEA), Government of KSA. Sixty male Wistar rats were randomly and equally distributed into six groups (*n* = 10).

**Group 1** received only a standard pellet diet.
**Group 2** received an oral prophylactic dose of 2 ml of AJDAE (0.75 mg/kg bw) daily using intragastric gavage for 30 days according to Vayalil (2002) and Mubarak et al. (2018b) with modifications.
**Group 3** received an oral prophylactic dose of 4 ml of AJDAE (1.5 mg/kg bw) daily using intragastric gavage for 30 days.
**Group 4** was intraperitoneally injected with a single dose of DOX (15 mg/kg, i.p.) at the end of the 28th day of the study to induce a nephrotoxic injury (Ellison, 2002).
**Group 5** was intraperitoneally injected with a single dose of DOX (15 mg/kg, i.p.) at the end of the 28th day of the study and received 2 ml of AJDAE (0.75 mg/kg bw) daily for 30 days using intragastric gavage according to Vayalil (2002) and Mubarak et al. (2018b).
**Group 6** was intraperitoneally injected with a single dose of DOX (15 mg/kg, i.p.) at the end of the 28th day of the study and received 4 ml of AJDAE (1.5 mg/kg bw) daily for 30 days using intragastric gavage according to Vayalil (2002) and Mubarak et al. (2018b).

### 2.6 Sample collection

Rats were left fasting 12 h before sampling and decapitation. Twenty‐four hours after intraperitoneal injection of DOX, rats were anesthetized using pentobarbitone sodium (60 mg/kg), and then, blood specimen from each rat was withdrawn via the optic vein, saved in a centrifuge tube, and remained at room temperature for 20 min. Sera were obtained by centrifuging tubes at 4500 *g* for 10 min using cooling centrifuge. Serum samples were utilized for determination of serum urea, creatinine, calcium, phosphorous, and uric acid by direct colorimetric method. Then, each rat's abdomen was dissected; the left kidney was excised and split up into three specimens. One kidney specimen was submerged instantly into 10% buffered solution of neutral formaldehyde and handled for histopathological inspection, the second kidney specimen was utilized for DNA extraction, and the third kidney specimen was employed for preparation of kidney tissue homogenate.

### 2.7 Preparation of kidney tissue homogenates

The homogenization of left kidneys' tissue was performed instantly following kidney tissue excision in a Teflon‐glass homogenizer. The kidney tissue specimens were maintained at 2–8°C in a bucket containing ice. A 200 mg weight kidney tissue specimen was excised from the left kidney of each studied rat and submerged in 2 ml of PBS/1 mM EDTA. The tissue specimens were homogenized completely and kept for one round of freezing at −80°C in a deep freezer. Using a cooling centrifuge, the homogenate samples were centrifuged at 18,000 *g* (+4°C) for 30 min. The supernatants of the homogenized kidney tissue samples were assembled instantly, distributed in Eppendorf tubes, and preserved at −80°C ready for use (Sabbah et al., 2018).

### 2.8 Oxidative and antioxidative markers

The activities of superoxide dismutase (SOD), glutathione peroxidase (GPx), and kidney tissue homogenates were assayed according to the methodology of Madkour & Abdel‐Daim (2013) and Mubarak et al. (2018b). Glutathione‐S‐transferase (GST), glutathione reductase (GR), and catalase (CAT) kidney tissue homogenates were estimated according to the methods of Khan and Sultana (2009). Malonaldehyde (MDA) was estimated in the kidney tissue homogenates according to the method of Ohkawa et al. (1979) that was modified by Mubarak et al. (2018b).

### 2.9 Detection of genomic DNA of rats' kidney abnormality

For studying the kidneys' tissue genomic DNA integrity of all the studied rats and according to the purification protocol of total DNA, the DNA extraction of each rat's kidney tissue specimen was performed using the QIAGEN tissue extraction kit (Sabbah et al., 2018). Preparation of agarose gel of molecular biology grade (2% agarose gel in 1× TAE buffer) was arranged in accordance with Kumar Gothwal et al. (2007). Gel electrophoresis was conducted at 100 V constant potential difference for 1 h. DNA fragments were pictured by UVI tech. photo‐documentation system.

### 2.10 Pathological study

#### 2.10.1 Tissue preparation procedure

Histopathological evaluation was performed on all candidate rats in this study (control and tested groups) for renal tissue sections. All studied renal tissue samples were excised in compliance to the timings of the study design according to the planned scarification schedule, followed by fixation in 10% neutral buffered formalin solution, routinely processed, embedded in paraffin, sectioned at 4 μm thickness, and finally stained with hematoxylin and eosin (H&E).

#### 2.10.2 Hematoxylin and eosin staining procedure

Heating for 1 h in a 60°C oven preceded the staining step for tissue fixation on the slide. After xylene deparaffinization and rehydration in grades alcohol (absolute ethanol, 90% ethanol, and 70% ethanol), the kidney sections were stained with hematoxylin then further washed in running tap water until the sections were blue, followed by eosin staining. Slides were then dipped in 90% ethanol once, transferred to absolute alcohol. Finally, the sections were cleared in 2 changes of xylene, mounted using Canada balsam, and covered with clean glass slide covers.

#### 2.10.3 Evaluation procedure

Each representative H&E‐stained slide was thoroughly reviewed by the pathologist (LSS) at low power examination for screening as well as higher power magnification for further characterization. Any observed morphological alterations were recorded, and they were compared with the control group for reference evaluation. Histopathological findings were evaluated in a modified semiquantitative four‐tier scoring system focusing on glomerular injury, tubular cyst/cast formation, and interstitial inflammation, and other abnormal observations were also considered when encountered (Zheng et al., 2005). Microscopic variations were assessed as tabulated in Table 1. Finally, a comparative analysis of data was done.

**TABLE 1:** Semiquantitative evaluation score for nephrotoxicity among study groups

Glomerular injury, tubular cyst cast formation, atrophy or dysplasia, and interstitial inflammation	
0	No disease
1	1%–25% of tissue affected
2	26%–50% of tissue affected
3	51%–75% of tissue affected
4	76%–100% of tissue affected

### 2.11 In silico analysis

#### 2.11.1 Ligand preparation

The structures of GC‐MS identified bioactive compounds and the antioxidant silymarin which is used as a standard for molecular docking were obtained from NCBI PubChem database and NIST Chemistry WebBook. Energy minimization for the compounds was done by MOE software to remove clashes.

#### 2.11.2 Selection and preparation of proteins

Two proteins (NF‐κB‐DNA and human soluble epoxide hydrolase) were chosen, which have a linkage with the renal health (Liu, 2019; Tamada et al., 2003). The 3D structures of NF‐κB‐DNA (PDB ID:1NFK) and human soluble epoxide hydrolase (PDB ID:3ANS) were obtained from (RCSB) Protein Data Bank (PDB). The synthetic inhibitors and water molecules were removed from the proteins structures. Energy minimization, protonation, and addition of hydrogen atoms were performed for the 3D structures of proteins by MOE software. The active site residues were determined by MOE site finder according to previous publications (Kpemissi et al., 2019).

#### 2.11.3 Molecular docking

The molecular docking experiment was done to generate the best binding affinity between proteins and ligands. The proteins were set rigid, and ligands were flexible. Conformations of different ligands with target protein were generated, and the best docking pose with the least binding energy was selected for the prediction of the interacting residues and bond types using Discovery Studio (Biovia, 2017).

### 2.12 Statistical analysis

All obtained data analyses were stated as mean ± standard error by one‐way analysis of variance using SPSS 21. Illustrating the differences between the means, the *t*‐test of significance was verified and the difference was deemed statistically significant when *p* ≤ .05.

## 3 RESULTS

### 3.1 GC‐MS analysis of Ajwa date extract

**FIGURE 1:**
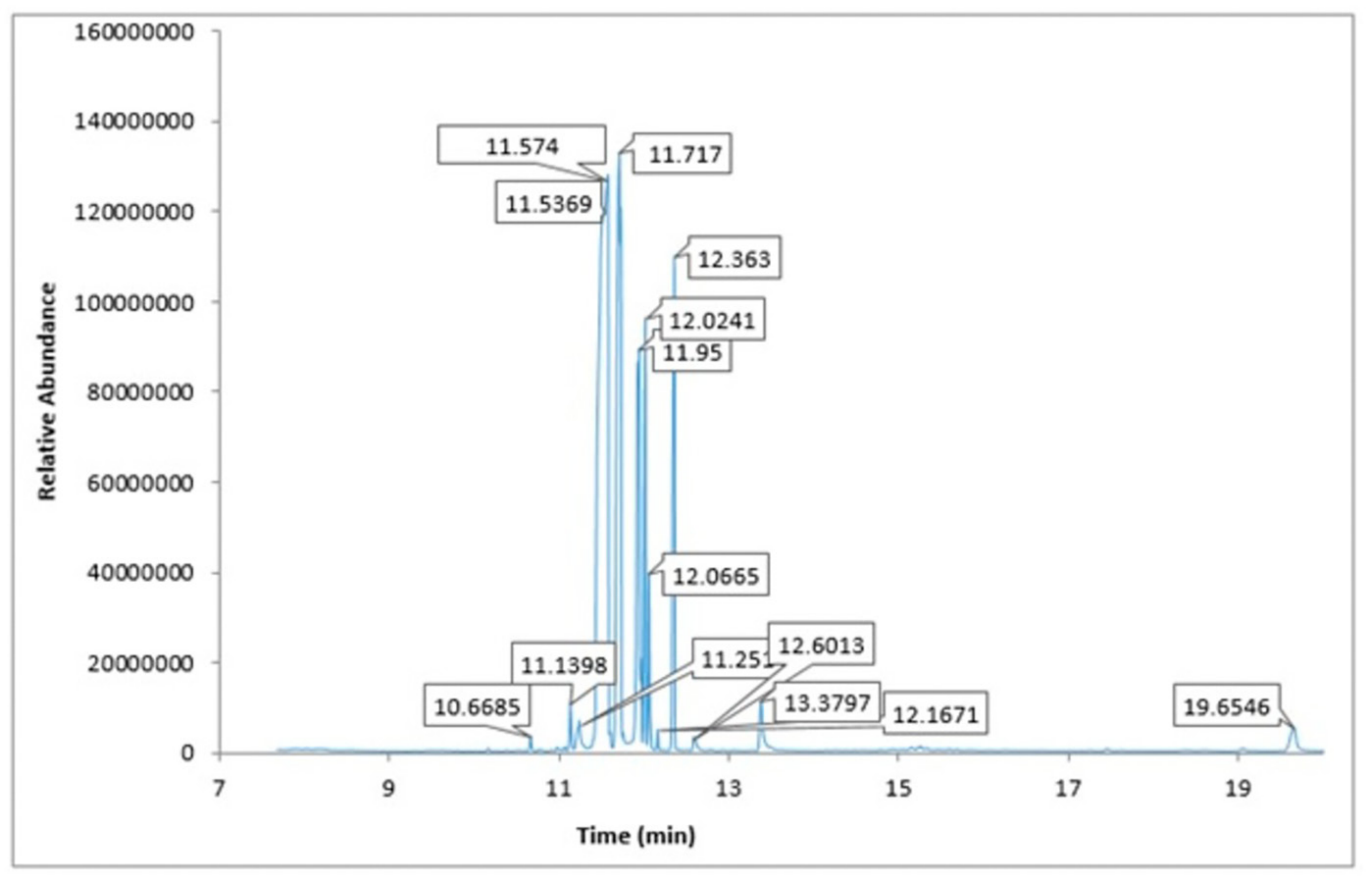
GC‐MS analysis of Ajwa date extract.

Of 39 phytoconstituents of Ajwa dates, 18 key compound peaks were acquired via pairing the ingredients' mass spectra with the NIST library, as shown in Figure 1. These compounds were glycerol, l‐threitol, 4TMS derivative, l‐(+)‐threose, tris(trimethylsilyl) ether, trimethylsilyloxime, d‐(+)‐Arabitol, 2‐pentenedioic acid, 2‐[(trimethylsilyl)oxy]‐,bis(trimethylsilyl) ester, d‐(−)‐tagatofuranose, pentakis(trimethylsilyl) ether (isomer 1), d‐Pinitol, pentakis (trimethylsilyl) ether, d‐Sorbitol, 6TMS derivative, d‐(+)‐Galactose, pentakis (trimethylsilyl) ether, pentafluorobenzyloxime (isomer 1), d‐Mannitol, 6TMS derivative, d‐glucopyranose, 5TMS derivative, l‐(+)‐Tartaric acid, 4TMS derivative, palmitic acid, TMS derivative, 3‐Heptadecen‐5‐yne, (Z), stearic acid, 9‐Octadecenoic acid, (E)‐TMS derivative, fumaric acid, di (2‐propylphenyl) ester, and α‐linoleic acid. Table 2 provides the identified compounds' chemical/formulae, M/Z ratio, molecular weight, peak area, and retention time.

**TABLE 2:** The chemical fingerprint of Ajwa date (AJD) extract shown by GC‐MS analyses

PK	RT (min)	Peak width (min)	Library/ID	Mwt	Chemical formula	M/Z
1	8.185	0.084	Glycerol	362.64	C_14_H_30_O_5_Si_3_	205
2	9.72	0.035	l‐Threitol, 4TMS derivative	410.22	C_16_H_42_O_4_Si_4_	73
3	10.192	0.02	l‐(+)‐Threose, tris(trimethylsilyl) ether, trimethylsilyloxime	423.84	C_16_H_41_NO_4_Si_4_	73
4	10.917	0.027	d‐(+)‐Arabitol	513.05	C_20_H_52_O_5_Si_5_	73
5	11.272	0.031	2‐Pentenedioic acid, 2‐[(trimethylsilyl)oxy]‐, bis(trimethylsilyl) ester	362.64	C_14_H_30_O_5_Si_3_	73
6	11.489	0.027	d‐(−)‐Tagatofuranose, pentakis(trimethylsilyl) ether (isomer 1)	541.06	C_21_H_52_O_6_Si_5_	73
7	11.611	0.024	d‐Pinitol, pentakis(trimethylsilyl) ether	555.08	C_22_H_54_O_6_Si_5_	73
8	12.087	0.029	d‐Sorbitol, 6TMS derivative	615.00	C_24_H_62_O_6_Si_6_	73
9	12.119	0.021	d‐(+)‐Galactose, pentakis(trimethylsilyl) ether, pentafluorobenzyloxime (isomer 1)	646.20	C_28_H_59_NO_6_Si_5_	73
10	12.156	0.024	d‐Mannitol, 6TMS derivative	615.25	C_24_H_62_O_6_Si_6_	73
11	12.336	0.023	d‐Glucopyranose, 5TMS derivative	541.06	C_21_H_52_O_6_Si_5_	204
12	12.49	0.04	l‐(+)‐Tartaric acid, 4TMS derivative	438.81	C_16_H_38_O_6_Si_4_	73
13	12.596	0.431	Palmitic acid, TMS derivative	328.60	C_19_H_40_O_2_Si	313
14	13.305	0.041	3‐Heptadecen‐5‐yne, (Z)	234.23	C_17_H_34_	79
15	13.491	0.034	Stearic acid	356.65	C_21_H_44_O_2_Si	117
16	13.385	1.3746	9‐Octadecenoic acid, (E)‐TMS derivative	354.6425	C_21_H_42_O_2_Si	73.0
17	15.6884	0.0359	Fumaric acid, di (2‐propylphenyl) ester	352.4236	C_22_H_24_O_4_	217
18	13.385	1.3746	α‐Linolenic acid	354.64	C_21_H_42_O_2_Si	75

Abbreviations: area Pct, peak area; Mwt, molecular weight; RT, retention time.

### 3.2 Group renal function profiles

Group 4 demonstrated a greater elevation (*p* < .01) in calcium, creatinine, phosphorus, serum urea, and uric acid than group 1 (Table 3). Additionally, comparison between the renal profiles' serum levels in groups 2 and 3 and the control group 1 was insignificant (*p* > .01). Further comparison with the rats administered with DOX showed significant constraint (*p* < .01) at both AJDAE levels for calcium (−31.78%, −31.71%), creatinine (−50.91%, −23.19%), phosphorus (−40.58%, −51.59%), serum urea (−24.36%, −39.9%), and uric acid (−24.35%, −36.43%). Also, comparison between the serum level fluctuations in groups 3 and 2 was insignificant (*p* > .01) (Table 3).

**TABLE 3:** Kidney profile of different studied groups

Groups	Urea, mg/dl	Creatinine, mg/dl	Ca, mg/dl	Ph, mg/dl	UA, mg/dl
Control	32.52 ± 3.74	0.24 ± 0.04	5.42 ± 0.28	5.95 ± 0.37	2.34 ± 0.40
AJDAE (0.75 g/kg bw)	30.35 ± 2.60	0.27 ± 0.04	5.52 ± 0.12	5.79 ± 0.41	2.32 ± 0.19
AJDAE (1.5 g/kg bw)	29.52 ± 3.02	0.28 ± 0.02	5.55 ± 0.12	5.63 ± 0.21	2.28 ± 0.30
DOX	50.43 ± 4.23***	0.59 ± 0.05***	11.63 ± 1.08***	8.76 ± 0.97***	3.87 ± 0.34***
DOX + AJDAE (0.75 g/kg bw)	38.14 ± 3.22^###^	0.35 ± 0.03^###^	5.71 ± 0.15^###^	6.73 ± 0.63^###^	2.64 ± 0.27^###^
DOX + AJDAE (1.5 g/kg bw)	34.43 ± 4.05^###^	0.35 ± 0.03^###^	5.63 ± 0.18^###^	6.63 ± 0.25^###^	2.46 ± 0.25^###^

*Note*: All data were characterized as mean ± SEM. Values were statistically tested using *t*‐test and significant differences at *p* < .05, *p* < .01, and *p* < .0001 as indicated by (*), (**), and (***) compared with normal control and (#), (##), and (###) compared with DOX‐treated group.

### 3.3 Antioxidant markers and LPO

**FIGURE 2:**
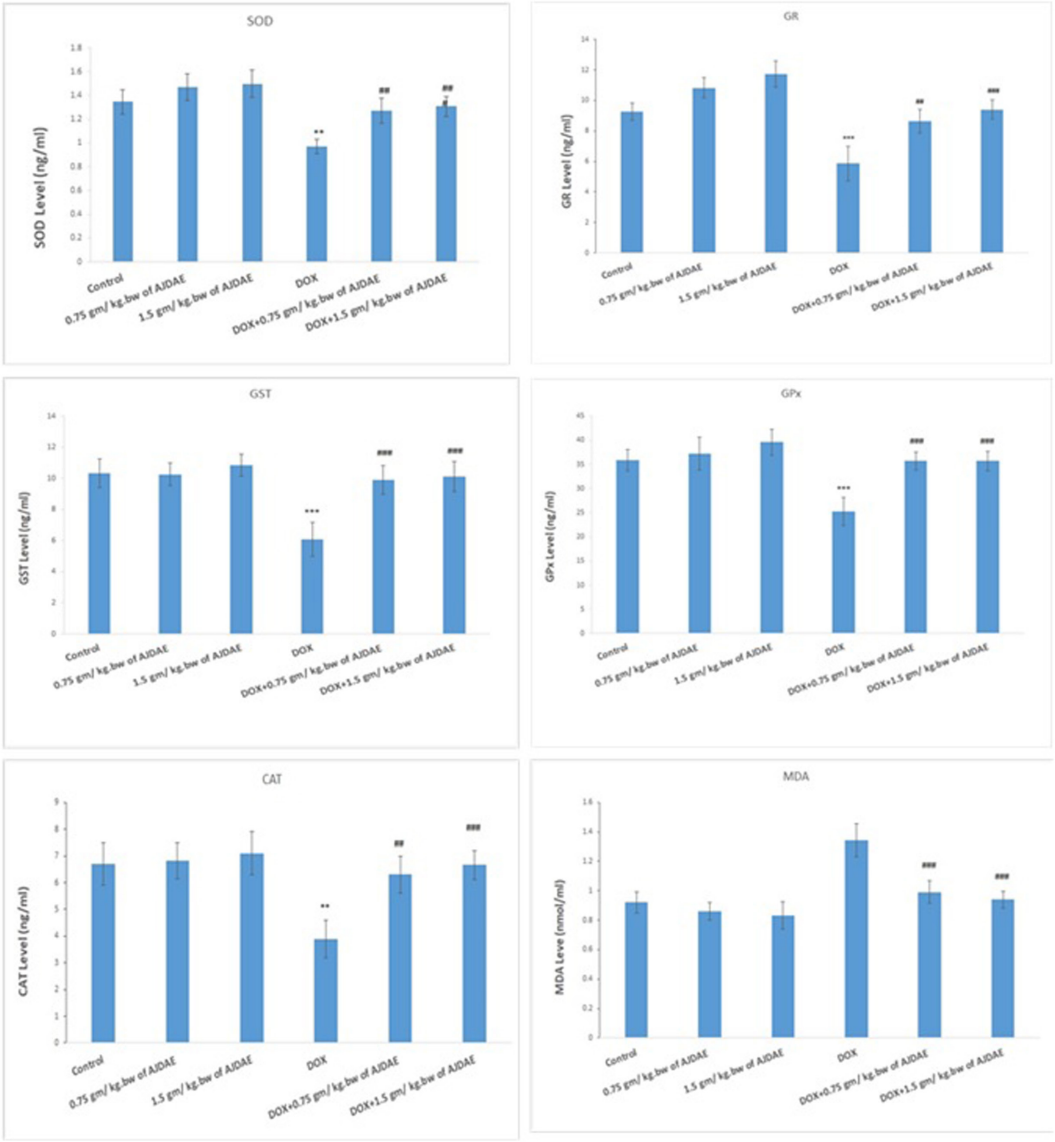
Effects of AJDAE on tissue SOD, GR, GST, GPx, CAT, and MDA levels in DOX‐treated rats. The values are mean ± SEM (*n* = 10). Statistical analysis was calculated via *t*‐test analysis. For estimation of *p* values, DOX‐treated group was compared with the control group, and AJDAE‐protected groups were compared with the DOX‐treated group. All data were characterized as mean ± SEM. Statistical data were tested using *t*‐test, and differences were expressed at *p* < .05, *p* < .01, and *p* < .0001 as indicated by (*), (**), and (***) compared with normal control and (#), (##), and (###) compared with DOX‐treated group.

Group 4 demonstrated significant depletion (*p* < .01) in the kidney homogenate levels of antioxidant markers (SOD, GR, GST, GPx, and CAT) and significant elevation (*p* < .01) of MDA levels in the kidney tissue homogenate compared with group 1 (Figure 2).

Fluctuations in the kidney tissue homogenate levels of antioxidant markers and MDA of groups 2 and 3 were insignificant (*p* > .05) when compared with group 1 (Figure 2).

When compared with the kidney tissue homogenate levels of group 4, those of groups 2 and 3 showed significantly increased (*p* < .01) depletion for SOD (31%, 63%), GR (41%, 62%), GST (26%, 35%), GPx (67%, 41%), and CAT (71%, 30%) (Figure 2). Further comparisons of the same groups revealed significantly decreased (*p* < .01) elevation of the kidney tissue homogenate levels of MDA (−47%, −60%).

Additionally, comparisons of the changes in the kidney tissue homogenate levels of SOD, GR, GST, GPX, CAT, and MDA of groups 3 and 2 were insignificant (*p* > .01) (Figure 2).

### 3.4 Electrophoretic pattern of the groups' DNA

**FIGURE 3:**
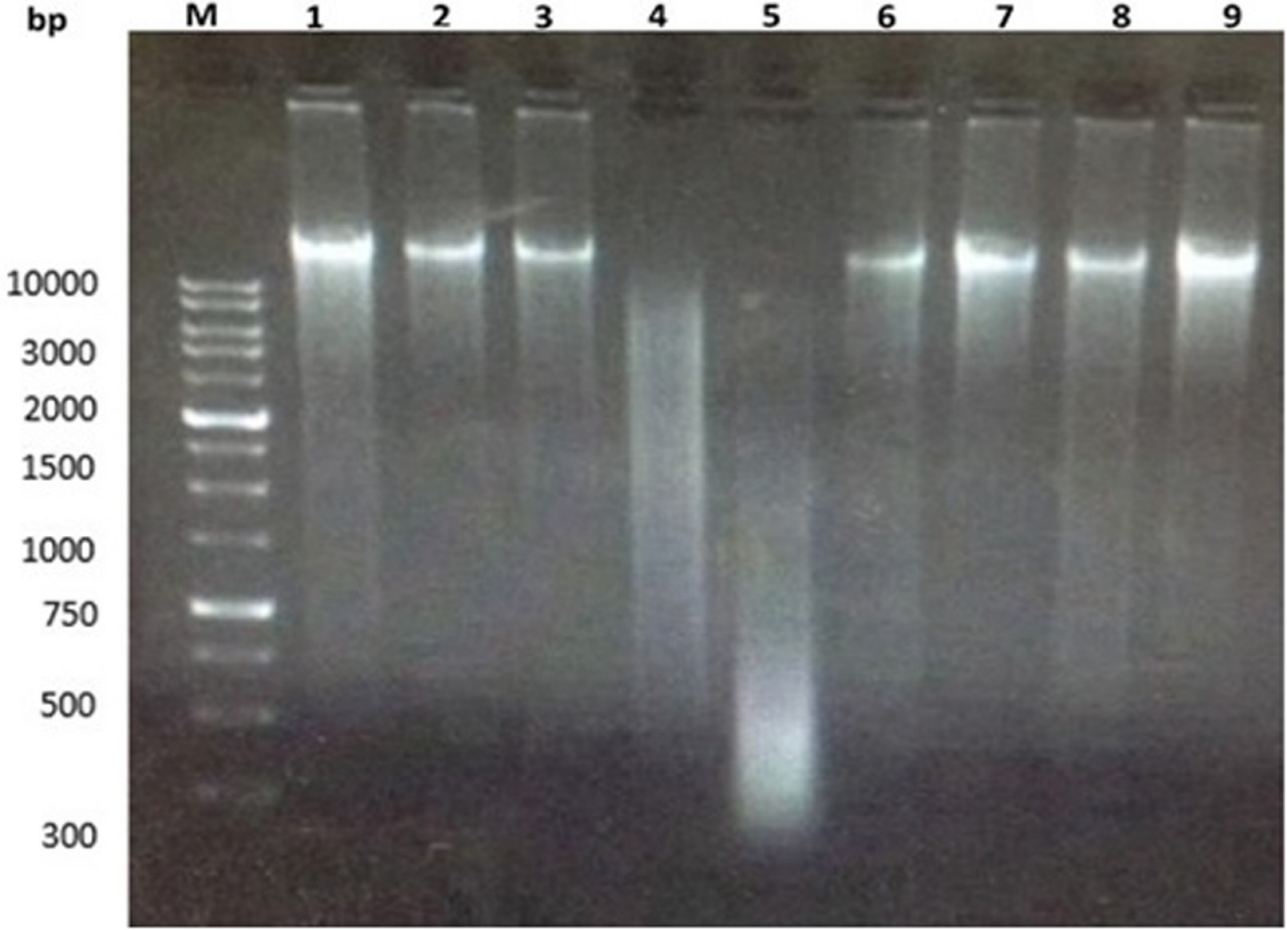
DNA fragmentation of rats treated with different concentrations of AJDAE following DOX‐induced nephrotoxicity. Lane M is a DNA marker with 10,000 bp. Lane 1 is normal group. Lane 2 is AJDAE group (0.75 g/kg bw). Lane 3 is AJDAE group (1.5 g/kg bw). Lanes 4 and 5 are fragmented DNA streaks (DOX‐treated group). Lanes 6 and 7 are DNA of rats' kidneys (0.75 g/kg bw of AJDAE + DOX group). Lanes 8 and 9 are DNA of rats' kidneys (1.5 g/kg bw of AJDAE protected + DOX group).

DNA extracted from the kidney tissues revealed a variety of banding forms (Figure 3). Group 1's genomic DNA presented a unique sharp band with no disintegration and tail formation. Groups 4's genomic DNA showed an entirely diverse style of banding; a classical band DNA fragmentation was identified that was not in Group 1. Groups 5 and 6 showed substantial kidney‐DNA recovery. Groups 2 and 3 did not exhibit any kidney tissue DNA disintegration.

### 3.5 Histopathology results

**FIGURE 4:**
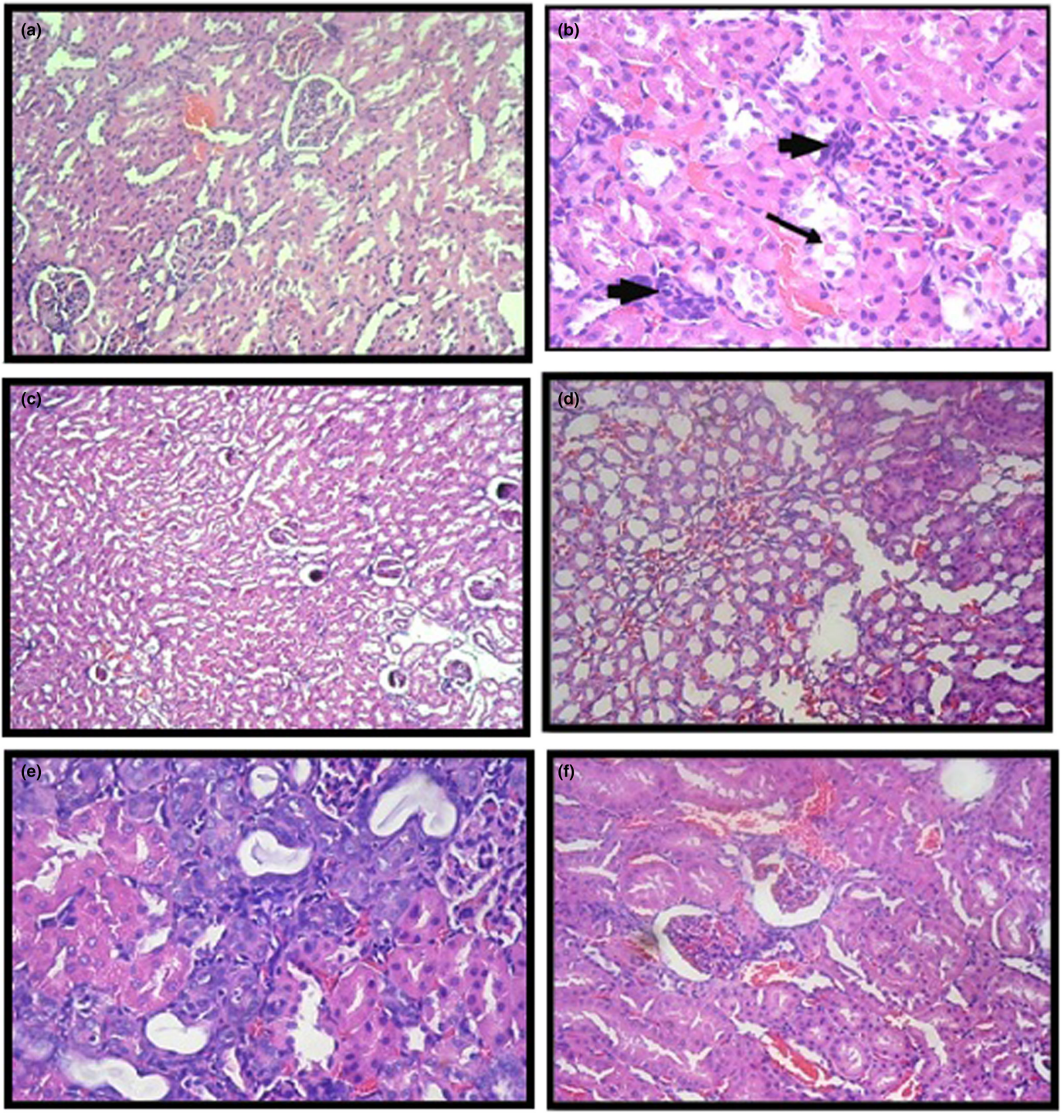
(a) Representative renal section in group 2 showing focal glomerular proliferation. Same changes were observed in group 3 (H&E × 400). (b) Renal section in group 4 showing interstitial inflammation (arrowhead) and focal tubular hyaline cast formation (arrow) (H&E × 400). (c) Renal tissue section in group 4 showing focal glomerular atrophy and sclerosis (H&E × 100). (d) Renal section in group 4 showing tubular cysts formation (H&E × 200). (e) Renal section in group 4 showing focal dysplastic tubular epithelium and pronounced nearby hyaline changes along with interstitial inflammation (H&E × 400). (f) Renal section in group 5 showing glomerular mesangial proliferation and tubular hyaline change (H&E × 400).

Some segments of group 1 were unexceptional and were established as reference tissue for the comparative evaluations. Segments of groups 2 and 3 displayed slight fluctuations, primarily in terms of endocapillary glomerular proliferation (Figure 4a). Group 4 tissue sections showed varying degrees of glomerular damage, such as mesangial hyperplasia and segmental sclerotic alterations (Figure 4b), and atrophic glomeruli alongside compensatory hyperplasia and neutrophilic glomerular proliferation (Figure 4c). Tubular changes encompassed hyaline change and cystic tubular atrophy (Figure 4d), but one segment demonstrated focal tubular epithelial dysplasia (Figure 4e). This entire group demonstrated remarkable effects in over 50% of the renal tissue sections investigated; only this group recorded a score of 4; the other five groups had no record. Measurably, the alterations in groups 5 and 6 were weaker than those in group 4. However, a score of 3 was recorded in a single specimen for each member of groups 5 and 6 (Figure 4f). Moreover, a minor swing toward normalcy was observed in group 6 compared with group 5, with a 100% increase in renal sections demonstrating <25% change. Conversely, the extreme pathological changes in renal tissue observed in group 4 (atrophy and dysplasia) were not present in groups 5 and 6. These adjustments were mesangial proliferation, some retained tubular hyaline fluctuations, and a lack of interstitial inflammation. Also the more grave injurious manifestations (cystic strophic dilation and tubular epithelial dysplasia) were exceptionally detected in group 4 (Table 4 **)**.

**TABLE 4:** Histopathological assessment data of all tested groups

Group	Renal tissue semiquantitative score					Total
	0	1	2	3	4	
	No. (%)	No. (%)	No. (%)	No. (%)	No. (%)	
Group 1	6 (60%)	4^a^ (40%)	–	–	–	10
Group 2	2 (20%)	6^a^ (60%)	2 (20%)	–	–	10
Group 3	6 (60%)	2^a^ (20%)	2 (20%)	–	–	10
Group 4	–	–	–	4 (40%)	6 (60%)	10
Group 5	–	2(20%)	7(70%)	1 (10%)	–	10
Group 6	–	4 (40%)	5 (50%)	1 (10%)	–	10
Total	14 (23.3%)	18 (30%)	16 (26.7%)	6 (10%)	6 (10%)	60

a Glomerular injury in groups 1 and 2 was exclusively manifested as endocapillary glomerular proliferation.

### 3.6 In silico studies

**FIGURE 5:**
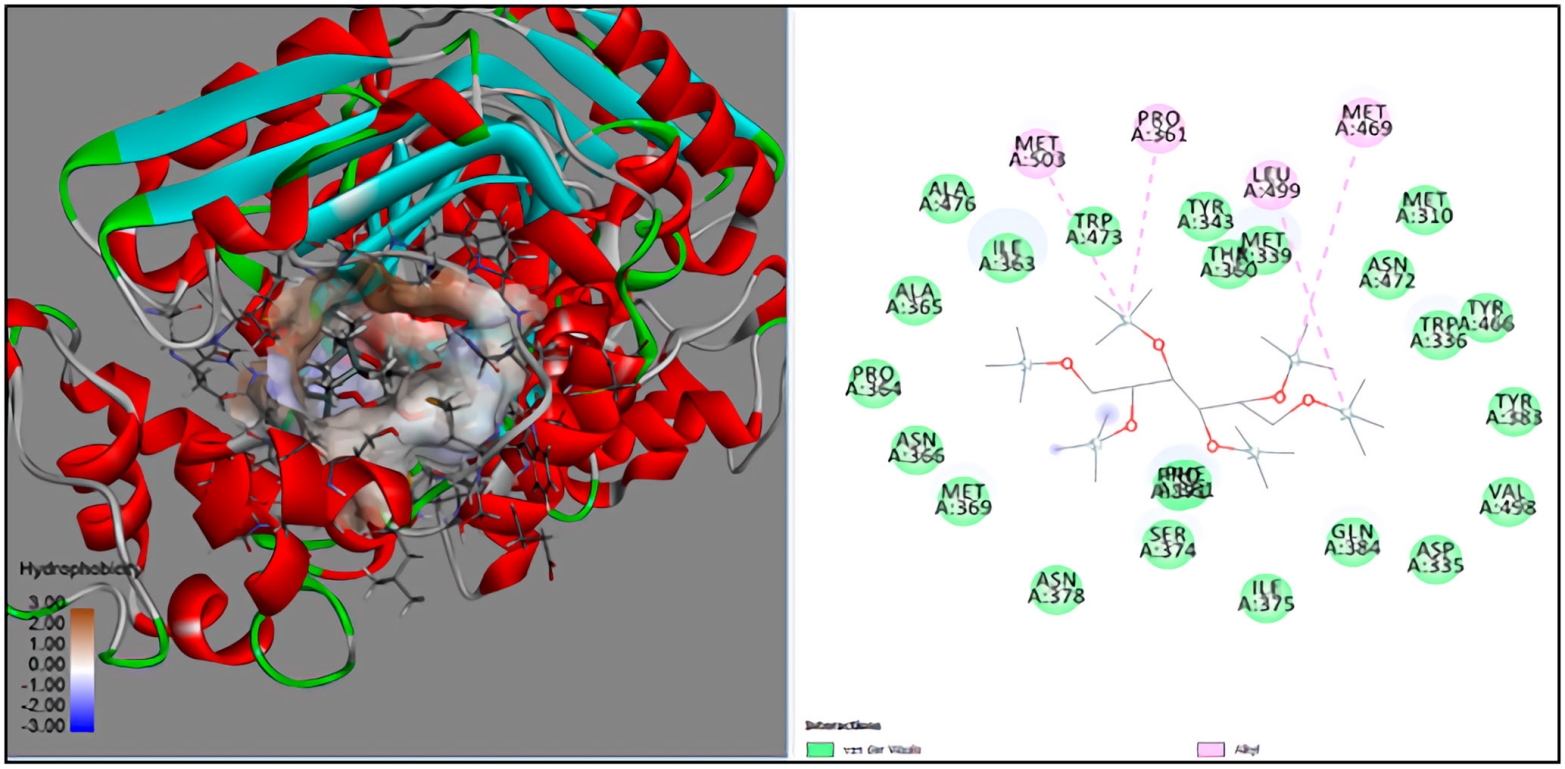
3D and 2D interaction of D‐Mannitol, 6TMS derivative, and human soluble epoxide hydrolase (PDB ID:3ANS).

**FIGURE 6:**
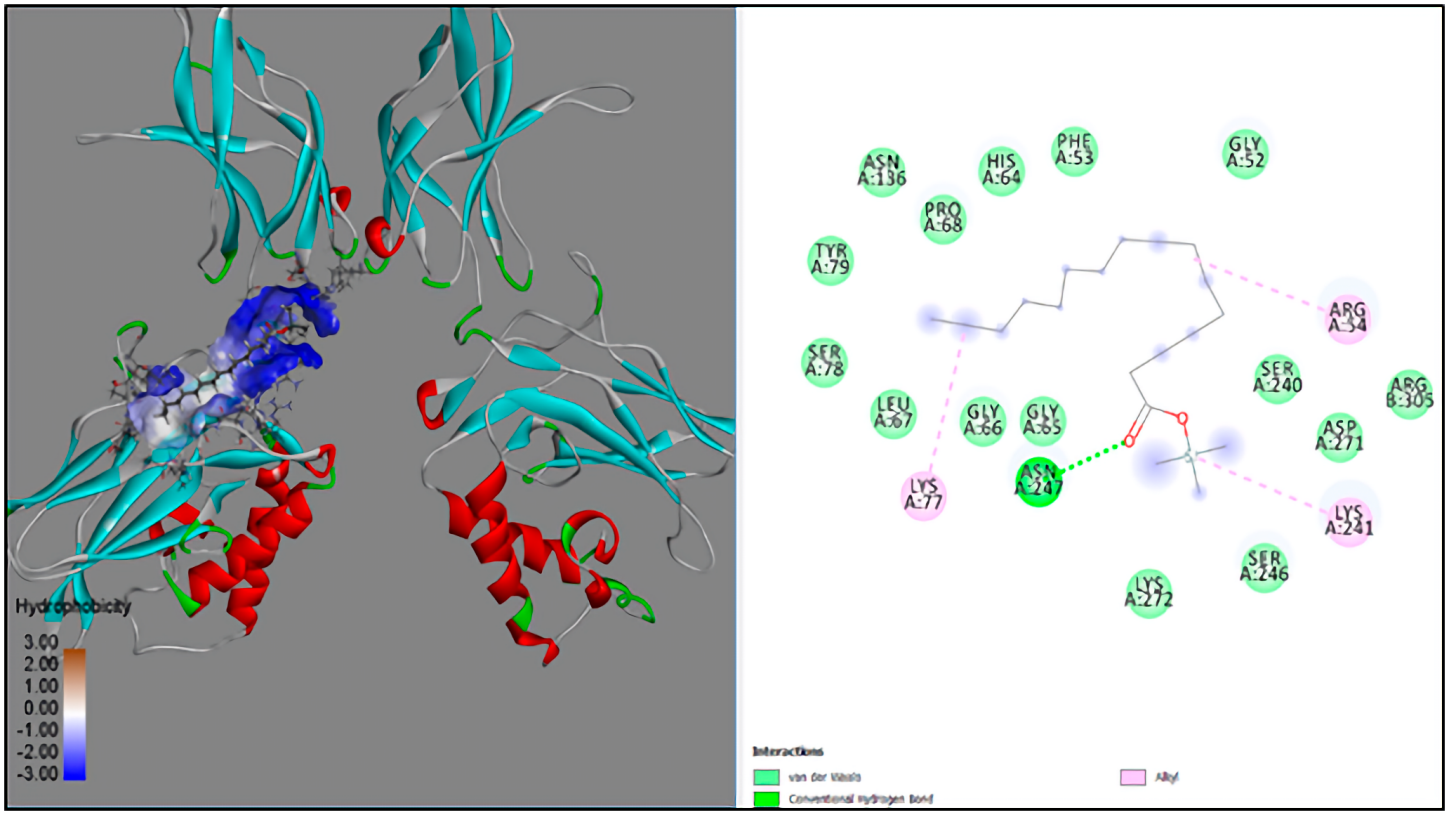
3D and 2D interaction of palmitic acid, TMS derivative, and NF‐κB‐DNA (PDB ID:1NFK).

GC‐MS analysis of proteins and bioactive compounds' interaction showed that these ligands have diverse binding affinity to the chosen proteins. The strongest binding affinity was noted between the D‐Mannitol, 6TMS derivative, and the human soluble epoxide hydrolase (PDB ID:3ANS) protein (Figure 5). Additionally, the top docking energy of −7 kcal/mol was observed in palmitic acid and TMS derivative, with NF‐κB‐DNA (PDB ID:1NFK) (Figure 6 and Table 5).

**TABLE 5:** The 2D structure and docking energy of the bioactive compounds

No.	Name	ID	2D structure	3ANS	1NFK
1	Glycerol	PC‐CID: 753	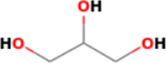	−4.3	−4
2	l‐Threitol, 4TMS derivative	CAS‐RN: 32381‐52‐5	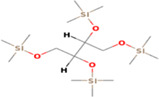	−8.8	−5.5
3	l‐(+)‐Threose, tris(trimethylsilyl) ether, trimethylsilyloxime	PC‐CID: 91696654	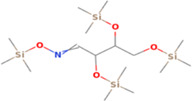	−8.9	−5.7
4	d‐(+)‐Arabitol	PC‐CID: 94154	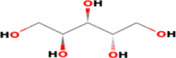	−5.2	−4.7
5	2‐Pentenedioic acid, 2‐[(trimethylsilyl)oxy]‐, bis(trimethylsilyl) ester	PC‐CID: 5366622	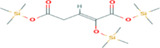	−10	−5.8
6	d‐(−)‐Tagatofuranose, pentakis(trimethylsilyl) ether (isomer 1)	PC‐CID: 523972	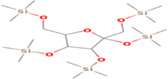	−7.9	−6
7	d‐Pinitol, pentakis(trimethylsilyl) ether	CID: 14534973	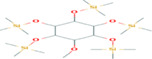	−9.9	−5.6
8	d‐Sorbitol, 6TMS derivative	CAS‐RN:	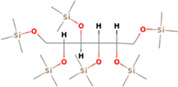	−10.8	−6.1
9	d‐Mannitol, 6TMS derivative	CAS‐RN:14317‐07‐8	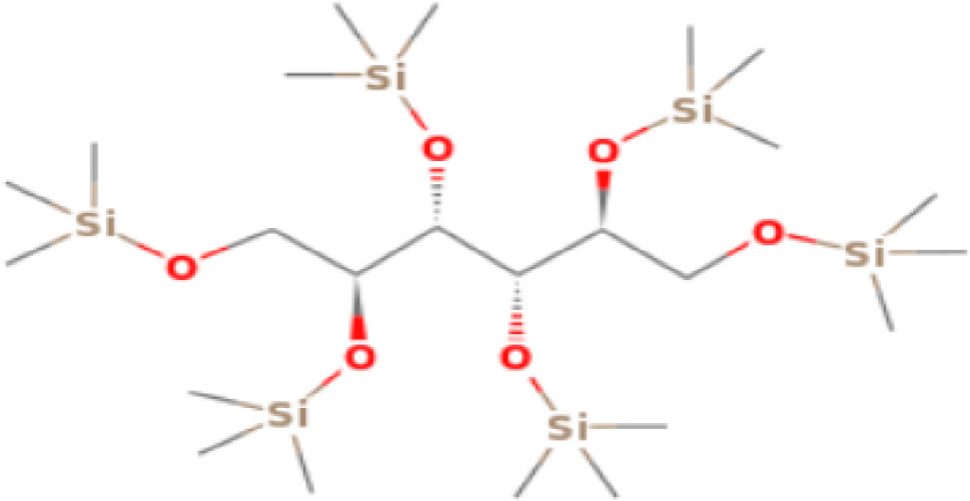	−10.9	−6.4
10	Beta‐d‐Glucopyranose, 5TMS derivative	CAS‐RN:2775‐90‐8	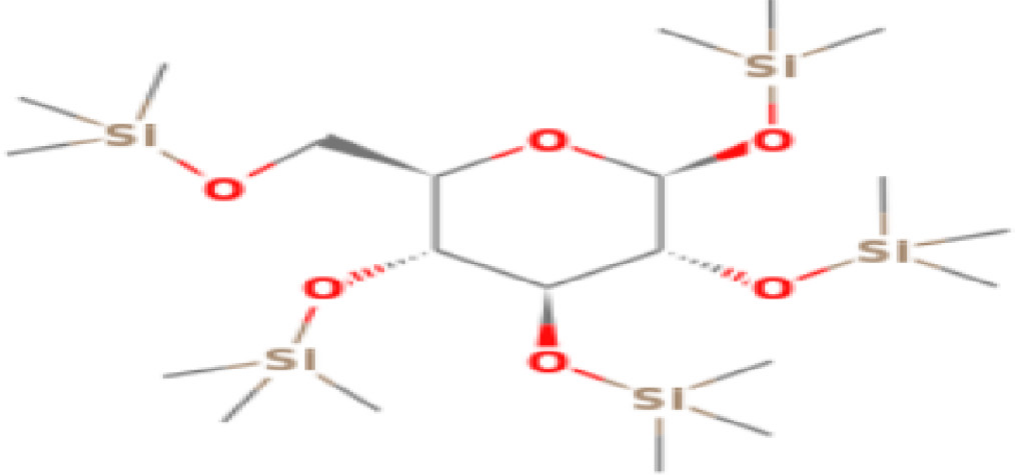	−6.4	−6.1
11	l‐(+)‐Tartaric acid, 4TMS derivative	PC CID: 519591	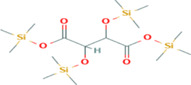	−9.2	−5.9
12	Palmitic acid, TMS derivative	CAS‐RN: 55520‐89‐3	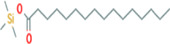	−8.5	−7
13	3‐Heptadecen‐5‐yne, (Z)‐	PC‐CID: 5367448	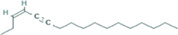	−7.3	−6
14	Stearic acid	PC‐CID: 5281	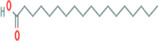	−8	−6.2
15	9‐Octadecenoic acid, (E)‐TMS derivative	CAS‐RN: 96851‐47‐7	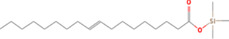	−7.4	−6.8
16	Fumaric acid, di (2‐propylphenyl) ester	PC‐CID: 91735211	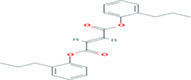	−6.4	−6.3
17	Silymarin (Standard)	PC‐CID: 5213	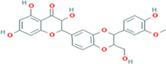	−7.8	−6.6

Abbreviations: CAS‐RN, Chemical Abstracts Service Registration Number; PC‐CID, PubChem Compound ID.

## 4 DISCUSSION

This study examines the AJDAE's nephroprotective impact on DOX‐induced nephrotic harm associated with oxidative stress, production of free radicals, and histopathological studies, confirming that an oxidative stress injury causes such effects.

This study verified DOX‐induced nephropathy, as evidenced by a significant increase in the levels of serum of urea, calcium, creatinine, phosphorus, and uric acid and supported by toxic histopathological fluctuations in noncontrol groups, providing quantitative and qualitative proof. For instance, group 4 administered with DOX without AJDAE protection demonstrated the highest nephrotoxicity score (4). The severe injuries (tubular epithelial dysplasia and cystic atrophic dilation) and interstitial inflammatory reactions occurred solely in this group. These findings correspond with existing research (Ayla et al., 2011; Refaie et al., 2016).

The GC‐MS results underscore that AJDAE is comprised of several phytoconstituents with antioxidant activities, including fumaric, linoleic, palmitic, and stearic acids (Table 2). These results correspond with previous research maintaining that Ajwa date contains many quantities of the aforementioned acids, typified by their antioxidant activity (Al‐Farsi & Lee, 2008a, 2008b; Ashrafian et al., 2012; Hayes et al., 2010; Nehdi et al., 2010). The AJDAE GC‐MS results confirm octadecanoic acid, an effectual anti‐inflammatory tool. It is especially beneficial for subduing the pro‐inflammatory signaling that leads to decreases in cytokines expression and proinflammatory mediators and inflammatory‐related ailments (Kang et al., 2018). The most common saturated free fatty acid is palmitic acid, a significant anti‐cancerous tool and potentially beneficial for human breast cancer treatment (Zafaryab et al., 2019). This is consistent with docking results that found palmitic acid demonstrates a strong inhibitory effect on NF‐κB protein. Additionally, stearic acid can better protect cortical neurons against oxidative harm by improving the endogenous antioxidant enzymatic system (Wang et al., 2007).

According to GC‐MS results, α‐linolenic acid is an active AJDAE ingredient. It can decrease the pervasiveness of chronic renal disorders, and it contributes to re‐establishing a healthy renal function (Gopinath et al., 2011). It aids with the prevention and management of other ailments, including autoimmune disease, ischemic heart disease, and strokes (Calder & Yaqoob, 2009; Connor, 2000). Furthermore, it has been confirmed that α‐linolenic acid decreases renal oxidative stress, and a synergistic effect was observed in its isomers (Saha & Ghosh, 2013). Kidney impairment diagnoses are aided by key biomarkers, urea, and creatinine (Khan & Sultana, 2004; Mohan et al., 2010). The findings agree with previous research that reported elevations in renal biomarkers' serum values (creatinine, urea, and uric acid) are related to tubular blockade and weakened renal architecture (Afsar et al., 2020; El‐Sheikh et al., 2012). Intraperitoneal DOX‐injected rats demonstrated some renal function issues, verifying that this drug can avert tubular cells' protein synthesis or improve renal tissues' LPO and free radical production (Naqshbandi et al., 2012).

Orally administered AJDAE could lessen the nephrotoxic impact generated by intraperitoneal DOX injection through substantial decreases in the serum values of calcium, creatinine, phosphorus, urea, and uric acid, compared with the DOX‐administered groups' renal function improving toward a normal level. This is attributable to DPFs antioxidant capability and anti‐inflammatory impacts, which decreases DOX‐related oxidative stress, inflammation, and tissue harm. DPF aqueous extracts contain high concentrations of polyphenolic elements that help prevent kidney intoxication and significantly improve the elevated levels of creatinine and urea caused by a range of chemotherapeutic medications (Abdelaziz et al., 2015; Yasin et al., 2015).

The serological and biochemical results, confirmed at the histopathological scope, explained the AJDAE protection. Although groups 4, 5, and 6 continued to show DOX‐induced oxidative cell injury, the AJDAE showed a clear antidotal effect on the nephrotoxicity in groups 5 and 6 only, as confirmed by the degeneration in the injury scope (score 4 was not documented; most recorded at score ≤ 2). This highlights AJDAE's protective role in combatting DOX nephrotoxicity, which is possibly due to its antioxidant, anti‐inflammatory, and regenerative impact, as confirmed in the past studies (Al‐Asmari et al., 2020; Younas et al., 2020).

In this study, DOX intoxication in rats resulted in raised levels of serum uric acid, conflicting with the results of Salah et al. (2012), which showed a reduction in plasma uric acid following dimethoate poisoning in rats. Furthermore, the increased uric acid level signified vascular disease composed of thickened preglomerular arteries and spread of smooth muscle cell (Kang et al., 2002). Blood uric acid is a strong antioxidant that is highly effective in foraging singlet oxygen and free radicals (Ames et al., 1981). Renal oxidative injury could result in damaged renal function.

This study noted that intraperitoneal DOX injections induced nephrotoxicity in rats because of oxidative stress and production of ROS, resulting in substantial decreases in the values of antioxidant enzymes SOD, GR, GST, GPx, and CAT. Additionally, the MDA serum level was substantially elevated compared with the normal control, along with tubular atrophy and raised glomerular capillary permeability. These latter results correspond with other research proposing that kidney intoxication is due to LPO, with the ensuing biological macromolecules injury through iron‐dependent oxidative harm and degenerative changes in the kidney depend on doses amassed and length of treatment, as DOX metabolites are partly expelled via the kidney (Wang et al., 2019). Another mechanism for the potential DOX‐related kidney harm is the transforming of DOX to semiquinone free radical through NADPH‐cytochrome P‐450, which generates superoxide anion and hydroxyl radical that triggers LPO (Rashid et al., 2013).

A substantial reduction in the CAT level in the nephrotic tissue of the DOX‐intoxicated group was found compared with the control group, which agrees with previous research that documented decreased CAT antioxidant activity with DOX treatment in rats (Ayla et al., 2011).

Orally administered AJDAE generated substantial improvement in the antioxidants' enzyme activities, alongside a significant decrease in the kidneys' MDA levels. This agrees with other research that identified substantial improvement of antioxidant enzyme activities for SOD, CAT, GST, and GSH level (Abdelaziz et al., 2015; El‐Far et al., 2016; Sahyon & Al‐Harbi, 2020). The improvement was attributable to DPF extracts' effectiveness as an antioxidant (El‐Far et al., 2016).

AJDAE's positive effects could be due to its active bio‐constituents capacity to remove free radicals and prevent LPO. DPF can chelate superoxide and hydroxyl radicals and very effectively suppresses macromolecule harm, such as LPO and protein oxidation in vitro (Vayalil, 2012). Another in vitro study showed that the flavonoid glycoside content of date extract is effectual as an LPO inhibitor (Zhang et al., 2013). Additionally, various anthocyanins, flavonoids, and phenolic compounds were established as protective agents for the kidney because of LPO harm (Allaith, 2008; Pandey & Rizvi, 2009; Sandhar et al., 2011).

This study identified a classical band disintegration of genomic DNA in the DOX‐intoxicated rats, but not in the control group. Groups 5 and 6 showed substantial recovery in kidney DNA. Groups 2 and 3 did not show any kidney tissue DNA disintegrations. These molecular findings correspond with other research that found that intraperitoneal DOX‐injected rats resulted in a classical fragmentation of DNA band not identified in the control group (Afsar et al., 2020).

DPF date has proved effective in decreasing micronuclei reductions, which is widely considered an indicator of improved DNA restoration in cells, or due to cell death or apoptosis of extreme DNA harm (Vukicevic et al., 2004). A previous study showed that DPF extract affects cellular recovery speed by regenerating the injured DNA areas (Diab & Aboul‐Ela, 2012).

The current histopathological findings verified that AJDAE treatment restored the tissue damaged by DOX injection, which agrees with recent research (Wang et al., 2019) in which the histopathological investigation of the kidney showed damaged tissue following DOX injection. Furthermore, microscopic scrutinization of tissue sections identified substantial enhancement in rats administered with AJDAE by increasing doses of 1 and 1.5 g.

Molecular docking established that d‐Mannitol and 6TMS derivative demonstrated high activity (docking energy recorded at −10.9 kcal/mol) on soluble epoxide hydrolase (sEH) enzyme. This enzyme is common in human tissues and is responsible for hydrolysis in many (Enayetallah et al., 2004), including renal tissues (Parrish et al., 2009). Additionally, as the GC‐MS process established, most bioactive compounds present in Ajwa extract demonstrated stronger binding affinity with human soluble epoxide hydrolase than the regular Silymarin (−7.8 kcal/mol).

## 5 CONCLUSIONS

The findings show that AJDAE can combat DOX toxicity; improve the renal histology and serum benefits of calcium, urea, creatinine, phosphorous, and uric acid; and stop further renal injury. AJDAE's protective effect could be attributable to its antioxidant impact through free radical chelating actions, declining inflammation, and membrane‐stabilizing activity. Potentially, Ajwa dates are therapeutically valuable for tackling doxorubicin nephrotoxicity.

## CONFLICT OF INTEREST

The authors declare no conflict of interest.

## ETHICAL STATEMENT

Ethical approval for this research was obtained from the Research Ethics Committee of Faculty of Medicine, King Abdulaziz University (reference no. 442‐16). The rats received animal care according to the guidelines of the Committee for the purpose of Control and Supervision of Experiments on Animals, KSA.

## Data Availability

All data analyzed during this study are included in this published article.
